# Protein Docking by the Underestimation of Free Energy Funnels in the Space of Encounter Complexes

**DOI:** 10.1371/journal.pcbi.1000191

**Published:** 2008-10-10

**Authors:** Yang Shen, Ioannis Ch. Paschalidis, Pirooz Vakili, Sandor Vajda

**Affiliations:** 1 BioMolecular Engineering Research Center, Department of Biomedical Engineering, Boston University, Boston, Massachusetts, United States of America; 2 Center for Information and Systems Engineering, Boston University, Boston, Massachusetts, United States of America; 3 Division of Systems Engineering, Boston University, Boston, Massachusetts, United States of America; 4 Department of Electrical and Computer Engineering, Boston University, Boston, Massachusetts, United States of America; 5 Department of Mechanical Engineering, Boston University, Boston, Massachusetts, United States of America; University of California San Francisco, United States of America

## Abstract

Similarly to protein folding, the association of two proteins is driven by a free energy funnel, determined by favorable interactions in some neighborhood of the native state. We describe a docking method based on stochastic global minimization of funnel-shaped energy functions in the space of rigid body motions (*SE*(3)) while accounting for flexibility of the interface side chains. The method, called semi-definite programming-based underestimation (SDU), employs a general quadratic function to underestimate a set of local energy minima and uses the resulting underestimator to bias further sampling. While SDU effectively minimizes functions with funnel-shaped basins, its application to docking in the rotational and translational space *SE*(3) is not straightforward due to the geometry of that space. We introduce a strategy that uses separate independent variables for side-chain optimization, center-to-center distance of the two proteins, and five angular descriptors of the relative orientations of the molecules. The removal of the center-to-center distance turns out to vastly improve the efficiency of the search, because the five-dimensional space now exhibits a well-behaved energy surface suitable for underestimation. This algorithm explores the free energy surface spanned by encounter complexes that correspond to local free energy minima and shows similarity to the model of macromolecular association that proceeds through a series of collisions. Results for standard protein docking benchmarks establish that in this space the free energy landscape is a funnel in a reasonably broad neighborhood of the native state and that the SDU strategy can generate docking predictions with less than 5 Å ligand interface *C_α_* root-mean-square deviation while achieving an approximately 20-fold efficiency gain compared to Monte Carlo methods.

## Author Summary

Protein–protein interactions play a central role in various aspects of the structural and functional organization of the cell, and their elucidation is crucial for a better understanding of processes such as metabolic control, signal transduction, and gene regulation. Genomewide proteomics studies, primarily yeast two-hybrid assays, will provide an increasing list of interacting proteins, but only a small fraction of the potential complexes will be amenable to direct experimental analysis. Thus, it is important to develop computational docking methods that can elucidate the details of specific interactions at the atomic level. Protein–protein docking generally starts with a rigid body search that generates a large number of docked conformations with good shape, electrostatic, and chemical complementarity. The conformations are clustered to obtain a manageable number of models, but the current methods are unable to select the most likely structure among these models. Here we describe a refinement algorithm that, applied to the individual clusters, improves the quality of the models. The better models are suitable for higher-accuracy energy calculation, thereby increasing the chances that near-native structures can be identified, and thus the refinement increases the reliability of the entire docking algorithm.

## Introduction

Genomewide proteomics studies, primarily yeast two-hybrid assays [1],[2] and high-throughput mass spectrometry [3],[4], provide a growing list of putative protein–protein interactions, and demonstrate that most if not all proteins have interacting partners in the cell. Elucidating the atomic details of these complexes requires further biochemical and structural information. While the most complete structural characterization of a complex is provided by X-ray crystallography, protein–protein hetero-complexes constitute less than 2% of protein structures in the Protein Data Bank (PDB) [5], and their number increases at a slow rate. In fact, many biologically important interactions occur in weak, transient complexes that will not be amenable to direct experimental analysis, even when both proteins can be isolated and their structures determined. Thus, there is substantial need for computational docking methods that can determine the structure of a complex from the separately solved structures of two component proteins.

Based on the thermodynamic hypothesis, at fixed temperature and pressure the Gibbs free energy of the macromolecule-solvent system reaches its global minimum at the native state of the complex. Thus, docking requires a computationally feasible free energy evaluation model and an effective minimization algorithm. It is expected that docking methods can utilize the rich set of modeling tools developed for predicting the structures of folded proteins. It has been established over the last two decades that the energy landscape of a foldable protein resembles a many-dimensional funnel with a free energy gradient toward the native structure [6]–[9]. A number of papers suggest that the landscape theory also applies to protein–protein association [10]–[12]. The size of the funnel is determined by the length scales of the long-range electrostatic and hydrophobic interactions and the geometry of the proteins, and hence the funnel is restricted to a neighborhood of the native complex [13]. There is a free energy gradient toward the native state, but the funnel is rough, giving rise to many local minima [14] that correspond to encounter complexes, some of which may be visited along a particular association pathway [15],[16].

While homology modeling approaches play an important role in protein structure prediction, most current docking methods are based on direct optimization, and attempt to find the global minimum of a function approximating the free energy of the complex. According to the results of CAPRI (Comparative Assessment of PRotein Interactions), a community-wide experiment devoted to protein–protein docking [17]–[20], the optimization involves either the systematic sampling of the discretized rotational/translational space using Fast Fourier Transforms [21],[22] or geometric hashing [23], or it relies on Monte Carlo (or Monte Carlo minimization) algorithms [24],[25]. Both optimization methods are generic, i.e., they do not rely on any assumption about the specific shape of the energy function to be minimized.

The use of special optimization methods that account for the funnel-like shape of the free energy function offers two potential advantages. First, being designed for minimizing funnel-like functions, such algorithms can be more efficient than generic approaches. Second, the success of such algorithms will be a stringent test of how well the funnel assumption describes the binding free energy landscape. This second point is particularly interesting, because protein–protein association occurs in the six-dimensional (6D) space of translations and rotations, at least for the classes of proteins whose backbones remain essentially unchanged upon association (e.g., many enzymes interacting with their inhibitors). Although the association is accompanied by conformational changes, these can be considered auxiliary, and the shape of the funnel can be studied over the entire conformational space. In contrast, the free energy of protein folding is defined in a substantially higher-dimensional space, and hence funnels can be generally studied only along some reaction coordinates [6]–[9].

### Minimization by Underestimation

**Figure 1:**
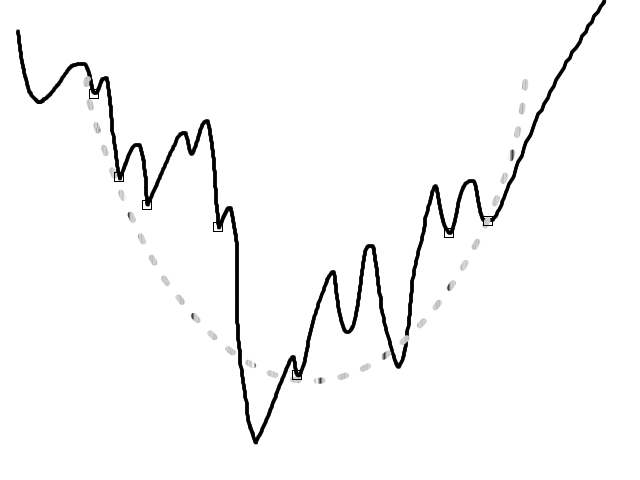
Funnel-like function and underestimator at a set of local minima indicated by small squares.

A minimization approach which is specific to funnel-like functions can be based on the concept of *underestimation*. The existence of a funnel implies that the free energy can be locally underestimated by a convex function (Figure 1). The original free energy function is extremely rugged with a huge number of local minima even in a small region of conformational space. Yet its convex underestimator is much smoother and still captures the overall funnel-like landscape, which provides a handle to free energy minimization. The quality of minimization through underestimation depends on the choice of underestimator functions, the way they are constructed and utilized to locate the global minimum, as well as how structured the free energy funnels are in conformational space. The Convex Global Underestimation (CGU) method [26] employed canonical quadratic functions as underestimators without any cross-terms. In that case the underestimator, based on a set of local minima, can be constructed by solving a Linear Programming (LP) problem. Uniformly distributed samples in the neighborhood of the underestimator's global minimum were then used to bias further sampling. The process was iterated with the set of local minima being updated, and the search region being reduced until certain convergence criteria are satisfied. CGU has been a very promising method with various applications in molecular structure prediction, including protein folding [27] and docking small molecules to proteins [28]. However, its restriction of using canonical quadratic functions limits its success in some cases [29], since the principal axes of the free energy surface are not necessarily aligned with the canonical coordinates. The performance further deteriorates as the dimensionality of the search space increases. We have used theoretical analysis to show and simple test problems to demonstrate that this restriction can lead to incorrect convergence [30].

Motivated by the potential advantages of underestimation and practical shortcomings of the CGU algorithm, we have recently extended the method using general quadratic underestimators and introduced biased sampling guided by the underestimator [30],[31]. Since the tightest underestimator in this class is obtained by solving a semi-definite programming problem, the method is termed SDU (Semi-Definite programming-based Underestimation). Semi-definite programming is computationally more demanding than the linear programming (still solvable in polynomial-time though) used in the CGU method. However, SDU typically requires fewer iterations and substantially improves optimization performance.

The SDU method starts from a set of *K* local minima **x** ^1^, …, **x** *^K^* of a funnel-like function *f*(**x**): 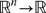 within a given region 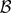 of the search space. Throughout the course of the algorithm we maintain a set 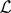 of local minima in the search region; initially 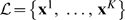. To capture the global funnel-like structure of *f*(**x**) within 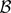 we construct a smooth (convex) quadratic function *U*(**x**) = **x**′**Qx**+**b**′**x**+*c*, where **Q** is a positive semi-definite matrix, 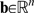, *c* is a scalar, and prime denotes transpose, such that *U*(**x**) underestimates *f*(**x**) at all local minima in 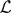, i.e., *U*(**x** *^i^*)≤*f*(**x** *^i^*) for all *i* = {1, 2, …, *K*}. The tightest possible underestimator (with an *L*_1_ norm metric) can be found by solving a semi-definite programming problem [30]. *U*(**x**) is a general convex quadratic function.

The underestimator *U* is used to guide further sampling. The minimum of *U*, denoted by **x** *^P^* and referred to as the *predictive conformation*, is in an energetically favorable region, and hence a new conformation can be generated by local minimization starting from **x** *^P^*. Additional conformations are obtained by local minimization with randomly generated starting points such that points in the vicinity of **x** *^P^* have a higher probability of being selected than points further away. To that end, we simply sample within 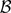 using a density function shaped as −*U*. The set 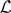 is being updated by adding these newly obtained conformations while removing unfavorable (i.e., higher energy) conformations, and the search area 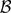 is being reduced to a neighborhood of **x** *^P^*. Using the updated conformations in 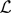 we repeat the underestimation step and the whole process is being iterated until a convergence criterion is met.

The SDU algorithm has a number of favorable properties when applied to funnel-shaped functions. Theoretical analysis shows [30] that **x** *^P^* converges in probability to the global minimum x* of the funnel-like function *f* as the number of samples *K* grows. When applied to test functions resembling the funnel-like free energy functions, SDU has been shown outperforming CGU and a simulated annealing algorithm which adaptively tunes its parameters, with much less required function evaluations and much higher success rates [30].

### Docking by Semi-Definite Underestimation

The main goal of this paper is to develop and test docking methods that use the SDU algorithm in order to find the global minimum of a funnel-like function approximating the free energy over regions of the conformational space. Over the last few years we have developed a multistage docking method that starts with rigid body search based on Fast Fourier Transform (FFT), selects and clusters 1000 to 2000 low energy docked structures, and retains the 10 to 30 largest clusters for further processing [32]. The conformational space is decomposed into separate regions defined by the clusters, where free energy attraction basins are believed to exist and the free energy landscape is assumed to be funnel-like. Then SDU is called upon to locate the global minimum within each region, by utilizing such funnel-like behavior.

As will be shown, in spite of the success of SDU as an optimizer for functions with funnel-shaped basins, its application to docking turned out to be far from straightforward. Although the method yields meaningful moves in either translational or rotational subspaces [30], minimization in the full 6D space of rigid body motions poses a challenge. This difficulty is well known in the robotics literature [33]. The Euclidean group *SE*(3), which is the semidirect product of 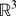 (translations) and the special orthogonal Euclidean group *SO*(3) (rotations), is a nonlinear manifold and its parametrization is critical to any optimization procedure [33]. In particular, we will show that the funnel-like shape of the free energy surface is affected by the parameterization of the search space, and that underestimation tends to fail unless appropriate parameters are selected.

We will describe two implementations of the SDU method: (*i*) SDU1, a cyclic coordinate descent strategy where rotational and translational moves alternate; and (*ii*) SDU2, a 5D strategy in which the distance of the two proteins is separately optimized and the SDU-driven search is performed in a lower dimensional space defined by 5 angular coordinates. Since the most energetically favorable distances occur when the two proteins are in contact but do not overlap, this strategy explores the free energy surface spanned by encounter complexes [15]. Results will show that the methods discover broad energy funnels, generate high quality docking predictions, and produce a substantial efficiency gain compared to Monte Carlo methods.

The comparison with Monte Carlo methods is based on our earlier preliminary work [34] where we have tested a method similar to SDU1 against a 10-protein set using reduced Gō-type potentials. Even with these relatively smooth potentials, underestimation in the full 6D space has not been effective, and we had to rely on optimizing in each (rotational or translational) subspace separately. In the present paper, we show that the SDU1 strategy is effective against a larger benchmark set using much more refined energy potentials. However, our main contribution is the introduction of SDU2 which is more effective and improves efficiency by a factor of 10 compared to SDU1. In addition, SDU2 provides interesting biophysical insights with its resemblance to docking by repeated micro-collisions.

## Results/Discussion

### Development of SDU-Based Docking Algorithms

To define the docking problem we fix the position and orientation of the first (receptor) protein. The six-dimensional vector **x** specifies the position and orientation of the second (ligand) protein. The variables **s** account for the side chain conformations in both proteins, and Δ*G*(**x**,**s**) denotes the free energy function. The minimization of Δ*G*(**x**,**s**) with respect to **s** is restricted to the side chains in the interface and is carried out by local methods. We define ΔG*(**x**) = Δ*G*(**x**,s*), where s* is an optimal solution; then the protein docking problem is finding the lowest minimum of ΔG*(**x**) in the region defined by the cluster, where **x** belongs to the space of rigid body motions, i.e., the Euclidean group *SE*(3). As described before, the parameterization of *SE*(3) is critical for optimization purposes, and hence we first describe our results concerning the parameterization of the search space.

The space *SE*(3) is the semidirect product of 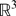 (translations) and *SO*(3) (rotations). The rotation group *SO*(3) is a 3-dimensional Lie group consisting of rotation matrices, i.e, 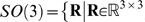, **RR**′ = **I**, det(**R**) = 1. The Lie algebra of *SO*(3), denoted by *so*(3), may be represented by the real skew-symmetric matrices
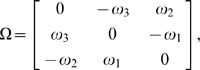
where 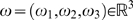. It is well-known that the one-parameter subgroups of *SO*(3), i.e., 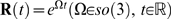 are geodesics [33],[35],[36], i.e., the shortest paths between two points on *SO*(3). Moreover, for **R**_0_, **R**_1_∈*SO*(3), Ω = log(**R**_0_′**R**_1_)∈*so*(3) and the distance between **R**_0_ and **R**_1_ can be defined by *ρ*(**R**_0_,**R**_1_) = ∥log(**R**_0_′**R**_1_)∥ = ∥*ω*_1_−*ω*_0_∥. This distance is a natural Riemannian metric on *SO*(3), i.e., it is bi-invariant with respect to the actions of the group (rotations). The exponential map from *so*(3) to *SO*(3) defined by Ω → **R**_0_ *e* ^Ω^ is a local diffeomorphism, i.e., there exist an open neighborhood of 0∈*so*(3) and an open neighborhood of **R**_0_∈*SO*(3) and an invertible, surjective, and smooth map from one neighborhood to the other whose inverse is also smooth. The local diffeomorphism induces a coordinate chart in a neighborhood of **R**_0_ that is known as exponential coordinate system. Given the definition of *so*(3) this coordinate system can be parameterized by 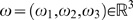.

Given the favorable properties of *SO*(3) (more generally *SO*(*n*)) such as the existence of a natural bi-invariant metric and, in particular, the simplicity of determining the geodesics of the manifold, the standard gradient based optimization algorithms on 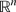 can be generalized for optimization on *SO*(*n*) [35]. However, generalization to the entire *SE*(3) is more difficult [33],[36]. Although *SE*(3) is also Lie group, its one-parameter subgroups are no longer geodesics. Moreover, *SE*(3) does not admit a natural (bi-invariant) Riemannian metric while its subspaces *SO*(3) and 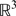 do [33],[36]. Attempting to implement SDU to *SE*(3) by simultaneous translational and rotational optimization, we have generally failed to construct useful underestimators. We have, however, found two strategies that were able to overcome this problem.

#### SDU1: Cyclic coordinate descent

A natural search strategy in *SE*(3) is to alternate between optimizing the free energy in *SO*(3) and in 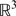 by a series of rotational and translational adjustments. As will be further discussed, the major disadvantage of this approach is that samples in one subspace cannot be reused in the other subspace, resulting in inefficient search.

#### SDU2: 5D search in the space of encounter complexes

The distance between the two proteins is separately optimized along the center-to-center vector, and the SDU search space is reduced to *S* ^2^×*SO*(3), where *S* ^2^ denotes the surface of the unit sphere in 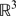. The receptor is fixed, with its center of mass placed at the origin of the coordinate system, and 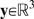 denotes the position of the center of mass of the ligand. In spherical coordinates **y** can be represented by (*r*,*θ*,*ϕ*), where *r* = ∥**y**∥, *θ* is the azimuth angle between the projection of **y** on the *xy*-plane and the *x*-axis (longitude, 0≤*θ*<2*π*), and *ϕ* is the zenith angle between the *z*-axis and the vector **y** (colatitude, 0≤*ϕ*≤*π*). The corresponding exponential coordinates are **σ** = (−sin *θ*·*ϕ*, cos *θ*·*ϕ*). The rotation of the ligand is described by the exponential coordinates 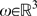 in *SO*(3).

In this coordinate system the free energy function is Δ*G*(*r*,**σ**,*ω*,**s**), where **s** describes the side chain conformations. Since **s** and *r*, respectively, are determined by local minimization and by a line search along the vectors connecting the centers of mass, by SDU we minimize the function
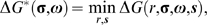
in the (**σ**,*ω*)-space. Thus, the SDU2 algorithm uses separate independent variables for side-chain optimization, center-to-center distance of the two proteins, and five angular descriptors of the relative orientations of the molecules. As will be shown, the removal of the center-to-center distance turns out to vastly improve the efficiency of the search, since the 5-dimensional space now exhibits a well-behaved energy surface suitable for underestimation. In addition, successive underestimators obtained during the course of the SDU2 algorithm can reuse the local minima obtained in the earlier steps, thereby reducing the number of required function evaluations. Moreover, SDU2 can use all conformations contained in the cluster to be refined, while SDU1 may use very few (or none) of these points as they may not lie in the subspaces explored. In fact, we have tested SDU1 for a 10-protein set with reduced energy potentials and compared its performance with a standard Monte Carlo method in [34]. SDU1 showed a modest speed-up factor of 2 compared with the Monte Carlo method, partly due to the issues mentioned above.

It is important that for any rotational state the energy is separately minimized along the vector connecting the centers of the two molecules. Since the lowest energy is generally attained at a distance that eliminates all atomic overlaps but retains some of the favorable van der Waals interactions, after minimization the two proteins are in contact with each other. Based on this property, the SDU2 algorithm essentially samples encounter complexes [15], resulting in meaningful energy values and efficient sampling. Simple arguments show that this parameterization is more natural than the sampling in the space of translations and rotations. In fact, the SDU2 strategy shows strong similarity to the model of macromolecular association in which translational diffusion brings the two proteins to a collision. Unless the enthalpy change of favorable interactions compensates for the free energy increase due to the loss of entropy, the proteins separate, rotate, and collide again. Thus, the conformational search proceeds in a series of “micro-collisions”, each resulting in an encounter complex [37].

### Test Results for SDU-Based Docking Algorithms

The SDU1 and SDU2 algorithms were tested on the protein pairs given in protein docking benchmark sets [38],[39] that contain enzyme–inhibitor, antigen–antibody, and “other” types of complexes, using the independently determined (unbound) structures of the component proteins. The algorithms were used to refine the top (most populated) clusters of docked structures generated by the rigid body docking program PIPER [22]. We consider the 10 largest clusters for enzyme–inhibitor complexes, and 30 clusters for antigen–antibody and “other” complexes. Table 1 shows results both from the rigid body docking and the SDU-based refinement procedures for all three types of complexes, each defined by its Protein Data Bank (PDB) code [5] in Column 1 of Table 1. We emphasize that for most complexes we docked the unbound (separately crystallized) protein structures rather than their bound conformations obtained by separating the complex. The exceptions are mostly a few antigen–antibody complexes for which no separate antibody structures were available and hence were taken from the complex. However, even in these cases we used the separately crystallized structure of the antigen as given in the benchmark sets [38],[39]. PDB codes for these “semi-bound” complexes are shown in bold italic fonts in Table 1.

**Table 1:** Docking and refinement results.

Complex^a^	PIPER			SDU1			SDU2			ZDOCK+RDOCK	
	Hits	RMSD	Rank	RMSD	Rank	*n̅*	RMSD	Rank	*n̅*	RMSD	Rank
**Enzyme–Inhibitor Complexes**											
1ACB	632	6.11	1	0.86	2	1425	2.78	3	93.8	5.66	1
1AVW	81	3.87	4	3.31	1	1381	3.54	2	90.0	2.50	1
1AVX	193	3.27	1	2.57	1	1393	4.18	1	91.2	2.62	39
1BRC	375	8.60	2	9.97	2	1431	4.50	2	98.5	8.92	1
1BVN	443	6.65	1	5.95	2	1498	1.11	1	81.7	1.78	8
1CGI	477	8.54	1	7.77	3	1425	2.40	3	100.0	9.03	16
1CHO	510	1.10	1	2.08	1	1475	2.64	2	91.4	5.25	1
1CSE	45	2.15	5	3.03	2	1340	5.99	2	97.3	2.83	6
1DFJ	30	6.59	4	3.80	2	1378	7.20	1	97.6	5.46	1
1E6E	55	6.88	9	4.85	1	1506	5.12	3	84.7	5.39	3
1EAW	114	2.02	3	3.03	2	1442	3.96	3	97.1	3.21	30
1MAH	171	1.80	3	1.37	1	1413	1.44	3	91.2	2.48	6
***1PPE***	605	4.24	1	2.42	1	1435	2.34	1	96.7	2.58	1
***1STF***	35	2.12	8	1.31	1	1303	0.80	1	92.7	1.70	4
1TGS	365	4.01	1	5.37	1	1343	6.02	1	98.0	6.18	158
1TMQ	25	3.03	9	1.01	2	1420	3.09	4	89.3	4.99	13
***1UDI***	217	4.37	1	1.73	2	1401	2.79	1	100.8	2.42	18
1UGH	97	4.75	3	4.86	1	1386	4.75	1	92.0	3.63	1
2MTA	161	5.30	1	6.47	3	1478	5.25	6	86.7	6.54	428
2PTC	322	7.49	1	7.37	4	1306	7.88	3	98.0	4.72	68
2SIC	76	7.76	4	1.93	1	1368	2.62	1	94.5	1.99	2
2SNI	103	8.56	2	8.71	1	1386	9.33	1	99.3	7.08	9
***2TEC***	235	1.89	1	1.56	1	1375	1.69	1	91.7	4.85	1
***4HTC***	73	5.21	7	4.82	3	1436	1.79	1	85.8	1.41	2
7CEI	178	7.68	1	6.61	3	1345	6.62	5	97.5	6.15	1
Average	224.7	4.96	3.0	4.11	1.76	1403	3.99	2.12	93.5	4.37	32.8
**Antigen–Antibody Complexes**											
1AHW	131	5.94	3	5.16	13	1163	2.83	4	98.7	3.08	2
1BVK	41	5.66	28	9.84	8	1249	5.80	29	96.0	6.34	435
***1EO8***	25	6.15	29	{11.60}	30	1194	2.50	6	97.3	N/A	N/A
***1FBI***	32	6.49	20	9.47	14	1212	3.43	17	92.7	7.97	266
***1IAI***	35	7.11	4	7.97	2	1210	6.20	4	96.7	7.90	212
***1MEL***	112	5.90	2	6.34	7	1239	2.65	4	100.0	3.52	103
1MLC	16	5.86	26	8.96	22	1242	7.57	19	93.3	9.50	47
***1QFU***	54	4.20	3	7.32	24	1091	2.54	22	90.0	2.97	96
1WEJ	26	3.65	19	5.45	19	1257	1.89	1	90.0	2.41	6
***2JEL***	136	6.74	4	5.40	6	1246	4.74	10	99.3	6.84	142
***2VIR***	14	8.90	25	8.50	28	1223	{11.12}	26	95.3	N/A	N/A
Average	56.6	6.05	14.8	7.82	15.7	1211	4.66	12.7	93.4	5.61	145.4
**Other Complexes**											
***1A0O***	193	6.01	2	6.91	1	1230	8.45	1	89.3	3.27	50
***1ATN***	272	6.57	1	6.38	4	1046	0.87	2	83.3	2.72	16
***1GLA***	396	8.38	1	5.65	3	1366	3.03	4	84.3	2.29	28
1MDA	60	7.59	15	{12.00}	13	1179	8.19	22	92.0	N/A	N/A
***1SPB***	252	3.41	3	1.80	1	1162	1.40	1	90.7	1.31	1
1WQ1	143	8.44	3	4.99	9	1164	10.00	1	93.3	4.88	656
***2BTF***	140	0.81	2	0.91	1	1165	1.68	1	86.0	2.50	1
2PCC	59	8.12	16	7.78	26	1168	9.96	1	93.6	N/A	N/A
Average	189.3	6.16	5.4	5.80	7.2	1185	5.44	4.1	89.1	5.62	125.3

a PDB codes for the bound-unbound docking problems are shown in italics. All others are unbound-unbound cases.

Columns 2–4 of Table 1 describe the docked complex structures generated by the rigid body docking [22] before any refinement. As described in the Methods, the PIPER docking program evaluates the energy for billions of docked conformations. We retain the 1000 best scoring structures, and cluster them using the pairwise RMSD as the distance measure and an optimally selected clustering radius. We have observed that the near-native structures tend to be in one of the largest clusters, and hence rank the clusters on the basis of their size. In fact, calculated for the rigid protein structures, the energy function is approximate, and better discrimination of the native structures can be achieved by focusing on the large clusters. The properties shown in Table 1 are the number of near-native conformations (or “hits” with less than 10 Å ligand interface *C_α_* RMSD) among the 1000 best scoring structures retained from the PIPER results, the Root Mean Square deviation (RMSD) between the native ligand structure and the docked structure at the center of the first cluster that includes a near-native conformation, and the rank of the particular cluster based on cluster size. Results are fairly good for enzyme–inhibitor complexes, as the 1000 structures, on the average, include over 200 hits, and the average RMSD is less than 5 Å between the native structure and the center of one of the three largest clusters. Although PIPER yields almost 200 hits for the “other” types of complexes, discrimination by cluster size is more difficult, and retaining the top 6 clusters results in 6.16 Å average RMSD. For antibody-antigen complexes PIPER generates much fewer hits, and on the average we have to retain 15 clusters to have a near-native structure in them.

The next 6 columns in Table 1 show the results for the SDU1 and SDU2 algorithms, in each case listing the RMSD for the lowest energy conformation in the first cluster that includes a near-native structure, the rank of the cluster, and the average number *n̅* of function evaluations. It is important to note that after the SDU refinement the clusters are ranked based on the energies of the SDU solutions rather than on cluster size. For comparison we also generated 2000 conformations (based on the original protocol) for each complex using version 2.3 of the rigid body docking program ZDOCK [21], and refined the structures using RDOCK [40], which performs local energy minimization. The last two columns of Table 1 show the RMSD of the first near-native structure found and its rank based on the RDOCK energy function.

For enzyme–inhibitor complexes both SDU1 and SDU2 give excellent results. On the average, the RMSD is reduced by almost 1 Å and the average rank is around 2. As the overall performance measure of the refinement we consider the number of complexes that have at least one prediction with less than 5 Å ligand interface *C_α_* RMSD from the native structure in the top 5 clusters. Such predictions would be termed “5 Å models” later for simplicity. As noted, the clusters of PIPER-generated structures are ranked based on their size (i.e., the number of structures). However, after the refinement the clusters are ranked based on the energy of their lowest energy structures. Among the PIPER-generated structures the top 5 clusters include less than 5 Å ligand interface RMSD predictions for only 11 of the 25 enzyme–inhibitor complexes. As shown in Table 1, both SDU1 and SDU2 increase this number to 17, i.e., a more than 50% improvement relative to the PIPER results. Notice that the 5 lowest energy predictions obtained by the ZDOCK/RDOCK procedure include 5 Å models only for 7 complexes. However, from the ZDOCK/RDOCK runs we retain low energy models, whereas the results provided by SDU1 and SDU2 are low energy clusters, and ranking clusters rather than individual structures generally removes some false positives, i.e., conformations that have low energy but are far from the native.

It is well known that antigen–antibody and “other” complexes are more difficult to predict than enzyme–inhibitor complexes [20]. For antibody-antigen pairs only the refinement by SDU2 improves the PIPER results. Although both the RMSD and the average rank of the first near-native cluster are reduced, at 12.7 the latter remains high (Table 1). Similarly, for the complexes in the “other” category only SDU2 improves both the RMSD and the rank. Even SDU2 yields only a total of seven 5 Å models in the top five clusters for antibody–antigen and “other” types of complexes. This result is somewhat disappointing, but note that docking by ZDOCK and refining by RDOCK leads to 5 Å models only for three complexes, and no near-native solution is found in four cases.

### Calculated Free Energy Surfaces

**Figure 2:**
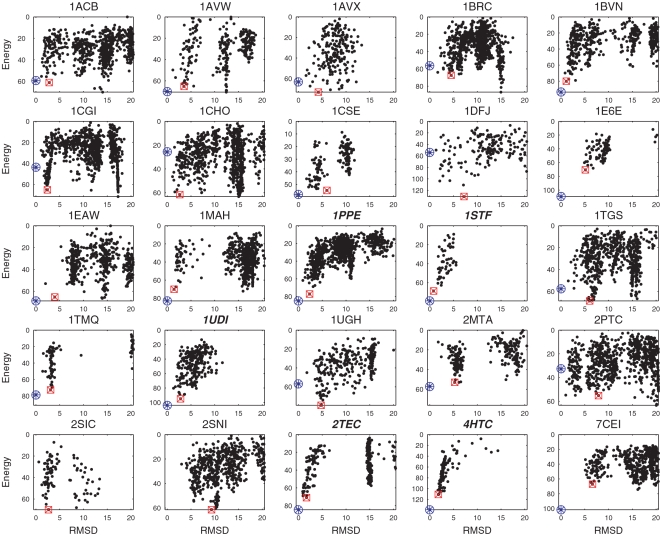
RMSD vs. energy plots for SDU2 sampled structures in the near-native funnel for enzyme–inhibitor complexes. The native structure is indicated by a blue circled asterisk and the SDU2 prediction by a red square.

Figure 2 shows the RMSD vs. calculated free energy for the 25 enzyme–inhibitor complexes. Again we show PDB codes for the “semi-bound” complexes in bold italic fonts. Each point represents a structure sampled in the process of refining the 10 largest clusters using the SDU2 algorithm. The encircled blue asterisk indicates the native structure and the first hit is shown as a red square. In spite of the one-dimensional representation of the energy function defined in the 6D rotational and translational space, the figure demonstrates the multi-funnel behavior over a relatively broad region (within 20 Å RMSD) of the native state. For most complexes the figure shows a well defined deep funnel within 5 Å RMSD from the native structure. According to Table 1, for 12 of the 25 complexes (including 5 “semi-bound” cases), this funnel is deepest among the 10 clusters sampled. For example, for 4HTC the near-native cluster is the 7th largest, but it is energetically the most favorable after refinement. For the remaining 13 complexes, clusters farther from the native structure yield the deepest funnels, resulting in false positive predictions. One of the worst behaviors can be observed for 2PTC with the first near-native cluster ranked 4, and Figure 2 shows a number of deep non-native funnels. However, on the average, one of the 3 deepest funnels is near-native (Table 1). This also shows the power of heuristic combination of entropic and enthalpic measurement, i.e., cluster size as a filter and refined cluster depth as ranking parameter.

**Figure 3:**
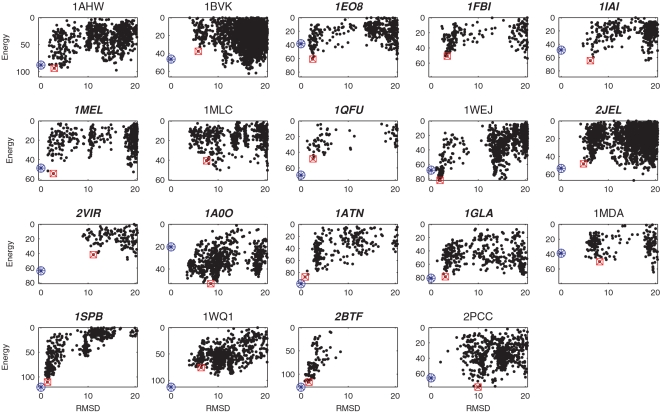
RMSD vs. energy plots for SDU2 sampled structures in the near-native funnel for antigen–antibody or other complexes. The native structure is indicated by a blue circled asterisk and the SDU2 prediction by a red square.

Figure 3 shows the RMSD vs. calculated free energy for the antigen–antibody and “other” complexes, sampled in the refinement of the 30 largest clusters by SDU2. All but 1SPB and 2BTF have multiple funnels, and the funnels are further from the native state than for enzyme–inhibitor pairs, demonstrating the well-known difficulty of estimating free energy using simple models, particularly for antigen–antibody complexes. Adjusting the conformation of interface side chains only by local minimization and keeping the backbone rigid also limits the accuracy of free energy calculation.

### The Effect of Space Selection on Search Efficiency

**Figure 4:**
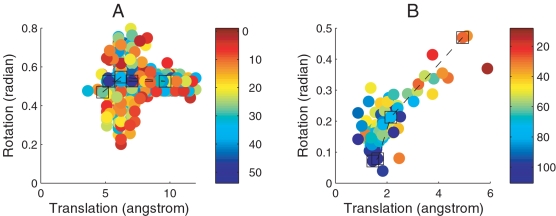
SDU1 (A) and SDU2 (B) trajectories in the near-native cluster for 4HTC. Translational and rotational distances plotted in the horizontal and vertical axes, are defined as ∥**r** ^1^−**r** ^0^∥ and ∥*ω* ^1^−*ω* ^0^∥, respectively, where (**r** ^1^,*ω* ^1^) denotes the position and orientation of the ligand and (**r** ^0^,*ω* ^0^) are the corresponding values in the native state. The points are color-coded depending on their energy (in Kcal/mol as shown on the right), and the points in the squares show the best local minimum in each iteration.

As shown in Table 1, the SDU2 strategy is much more efficient than SDU1, and also provides substantially better results for the more difficult problem of docking antigen–antibody and “other” complexes. These differences are demonstrated in Figure 4A and 4B that show, for the near-native cluster of the complex 4HTC, the conformations sampled and minimized by each algorithm. The horizontal and vertical axes, respectively, represent translational and rotational distances between each sampled ligand conformation and the one in the native structure, the latter placed at the origin of this coordinate system. The rotational distance here is defined as the length of the minimum geodesic (i.e., the minimum rotation in radian) between two rotations [36]. As discussed earlier, this distance between **R**_0_ and **R**_1_ can be defined by *ρ*(**R**_0_,**R**_1_) = ∥log(**R**_0_′**R**_1_)∥ = ∥*ω*_1_−*ω*_0_∥. The points are color-coded according to their energies, from low (blue) to high (red) energy structures.

As shown in Figure 4A, the separate treatment of translational and rotational subspaces in the SDU1 algorithm is highly inefficient, resulting in the sampling of many conformations with relatively high energies. Although all energy values shown are obtained by local minimization, the latter is unable to reduce the energy if the two proteins are too far from each other, which frequently occurs with the SDU1 algorithm. In addition, as indicated by the parallel lines, very similar translational regions are re-sampled at slightly different rotational coordinates, and vice versa. In contrast, as shown in Figure 4B, the SDU2 algorithm smoothly and efficiently descends toward the bottom of the free energy funnel. Based on the ranges of sampled free energy values, SDU2 sampled much lower energy regions compared to SDU1. Since the search is restricted to biophysically meaningful encounter complexes, the more consistent energy values facilitate the construction of better underestimators during the search. According to Figure 4B, for SDU2 there is a clear trend that structures closer to the native complex generally have lower energies, resulting in a deep and broad free energy funnel in this space. The existence of such free energy funnels is much less obvious, even in each separate subspace, when sampled by the SDU1 algorithm (Figure 4A).

In test docking problems even the slower SDU1 algorithm outperformed a standard Monte Carlo method by reducing the number of function evaluations by a factor of two [34]. As shown in Table 1, SDU2 further reduces the computational costs by *a factor of* 11 *to* 15, depending on the type of complex. Since most of the computational time is spent in energy evaluations, the computational gain of SDU2 over SDU1 is more than a factor of 10, and we estimate that SDU2 achieves more than 20-fold efficiency gain compared to Monte Carlo methods. We tested our algorithms on a 128-node biowulf cluster (IBM eServer×Series). Each node contains dual 1 GHz PIII processors with 2 GB memory. A typical refinement by SDU2 for each PIPER-generated cluster would take 2 to 6 processor-hours. The running time varies with the protein complex size (especially the interface size) and the number of iterations before convergence. Notice that on the average SDU2 samples only about 100 encounter structures for each cluster. No particular efforts have been made to accelerate either the interface side-chain search or the line search to determine the center-to-center distance. The CPU times can be compared to those reported for a server [41] based on the RosettaDock algorithm [42]. The server performs 1000 independent Monte Carlo simulations within 30 Å *C_α_* RMSD of a starting structure, as described in the RosettaDock protocol [42]. A typical run requires about 65 processor-hours.

### Repeatability of the SDU-Based Docking Algorithm

**Figure 5:**
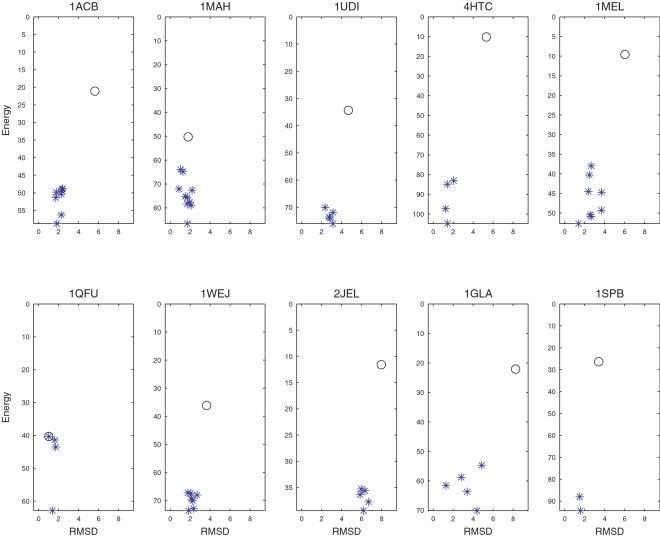
RMSD vs. energy plots for predictions from 10 independent SDU2 runs for the first near-native clusters of 10 randomly chosen protein complexes. The rigid body prediction before SDU2 refinement is indicated by a black circle and the SDU2 predictions by blue asterisks. (Note that some of the latter points overlap.)

SDU based docking algorithms are stochastic in nature. Although we gave a theoretical guarantee of probabilistic convergence to the global minimum of funnel-like functions under some fairly general conditions [30], practical protein docking problems do not necessarily satisfy all these conditions. To check the variations in our results we have repeatedly run SDU2 to refine the near-native cluster from PIPER (columns 3 and 4 in Table 1) for 10 randomly selected complexes, including 4 enzyme–inhibitor, 4 antigen–antibody and 2 “other” complexes. SDU2 was run 10 times independently for each complex with the same set of parameters. The results (ligand interface *C_α_* RMSD versus corresponding free energy values) are shown in Figure 5, where black circles represent the rigid body predictions from PIPER before refinement and blue asterisks represent the independent SDU2 predictions. Some of the latter overlap, resulting in less than 10 distinct solutions. SDU2 is able to lower the free energy values in all cases and to improve RMSD in most of them. Table 2 shows the mean (indicated by overline) and the standard deviation (indicated by *σ*) for both the ligand interface RMSD and the free energy for each complex. Although the average standard deviation is relatively large (about 4.4 Kcal/mol) for free energies, it is less than 0.5 Å for RMSDs. This result indicates a good level of robustness, considering that our free energy model does not discriminate among structures within 1 Å RMSD from each other. Note that the SDU-based algorithms rely more on the collective distribution of a set of encounter structures in the free energy funnel, rather than on a single low energy structure, which reduces the sensitivity of the results to the variations in the starting structures.

**Table 2:** Repeatability test for SDU2.

Complex	Rigid Body Prediction		SDU2 Predictions			
	RMSD	Energy	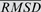	*σ_RMSD_*	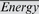	*σ_Energy_*
**Enzyme–Inhibitor Complexes**						
1ACB	5.63	−21.09	2.17	0.28	−51.15	3.50
1MAH	1.80	−50.22	1.60	0.43	−74.57	6.74
1UDI	4.66	−34.37	2.60	0.34	−71.58	2.19
4HTC	5.32	−10.22	1.84	0.34	−86.87	7.72
**Antigen–Antibody Complexes**						
1MEL	6.04	−9.53	2.67	0.65	−44.67	5.88
1QFU	1.02	−40.35	1.18	0.28	−43.03	7.06
1WEJ	3.65	−36.08	2.04	0.32	−69.02	2.41
2JEL	7.96	−11.61	6.08	0.26	−36.09	1.42
**Other Complexes**						
1GLA	8.27	−22.08	4.10	1.23	−58.23	5.33
1SPB	3.41	−26.31	1.51	0.03	−88.63	2.00
Average				0.42		4.42

### Conclusions

The successful application of the Semi-Definite programming-based Underestimation (SDU) search algorithm to protein–protein docking further validates the assumption that the free energy landscape of the complex is a funnel in some neighborhood of the native state. However, the direct application of SDU in the space *SE*(3) of rotations and translations fails to yield useful underestimators. Alternating searches in rotational and translational subspaces yields a feasible but inefficient algorithm. We have substantially improved performance by separately optimizing the center-to-center distance and describing *SE*(3) in terms of five angles. It is potentially important that this strategy samples encounter complexes, and hence it is reminiscent of the model of molecular association through a series of micro-collisions [37]. Results emphasize that the funnel-like shape of the free energy surface seen in this parameterization of *SE*(3) is largely lost when changing to the straightforward description of the space in terms of rotational and translational coordinates.

The underestimation approach has been used in the latest rounds of CAPRI with considerable success [19],[43], and it provides a promising platform for improving docking methods. We note that Marcia et al. [44] recently reported the application of SDU to the docking problem using the general quadratic underestimation method we have earlier developed [30],[31]. However, the central problem of parameterizing the search space was not discussed and the method was applied only to five bound docking problems using co-crystallized structures, which is much easier than docking two separately crystallized proteins. In fact, we believe that the separate minimization along the center-to-center vector and the use of five angular descriptors can improve the performance of any minimization algorithm used for docking.

## Methods

### Protein Docking Benchmark

The SDU1 and SDU2 algorithms were tested on complexes from the protein docking benchmark sets [38],[39]. These sets contain enzyme–inhibitor, antigen–antibody, and “other” types of complexes. As described, the algorithms were used to refine the separate clusters generated by the rigid body docking program PIPER. For enzyme–inhibitor complexes we refined only the 10 largest clusters and hence restricted consideration to complexes from the two benchmark sets for which these clusters included at least one near-native conformation. In addition, we disregarded four enzyme–inhibitor pairs that form oligomeric rather than binary complexes, resulting in the 25 test problems. The refinement algorithms were also applied to 11 antigen–antibody and 8 “other” type of complexes from the benchmark set 1 [38] that had at least one near-native structure in the 30 largest clusters. We emphasize that tests for most complexes involved separately determined protein structures as given in the benchmark sets [38],[39]. The exceptions are a few of the antigen–antibody complexes in which the antigen was separately solved but the antibody structure was taken from the complex.

### Rigid Body Docking

The rigid body docking program PIPER [22], based on the FFT correlation approach, systematically samples billions of docked conformations on a grid. Compared with other FFT-based approaches that use only shape complementarity and electrostatics for scoring, the scoring function in PIPER also includes the statistical pairwise potential DARS (Decoy As Reference States) [22]. Since the potential is represented as the sum of a few correlation functions through the eigenvalue-eigenvector decomposition of the matrix of the DARS interactions energy coefficients, the energy can be very efficiently evaluated using Fourier transforms. In conjunction with this higher accuracy scoring function, the PIPER program significantly enriches the hit rates among top ranked predictions for the benchmark sets described above.

For each complex, we retained the 1000 lowest energy predictions and clustered them using pairwise RMSD as the distance metric [45]. The resulting clusters were ranked based on their size, reflecting a preference for local minima with broad regions of attraction [32]. We have retained at most 30 clusters for each complex, each one being roughly 10 Å RMSD wide.

### The Semi-Definite Programming-Based Underestimation (SDU) Algorithm

#### Constructing an underestimator

We start with a set of K locally minimized structures 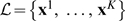 (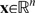, where *n* = 3 for SDU1 and *n* = 5 for SDU2) within each region 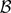 defined by a cluster and the corresponding free energy values Δ*G*. (The detailed free energy models would be described in the next part.) In this study, the set 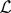 is initially chosen by the cluster center and (*K*−1) structures with the lowest PIPER scores within each cluster. When no cluster information other than its representative is available, this can be simply a set of locally perturbed structures around the cluster center. We are interested in constructing an underestimator which underestimates the free energy surface at those samples in set 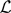 and captures the general funnel-like landscape. The family of underestimators used here are convex general quadratic functions 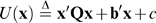, where 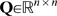, and *c* is a scalar.

Using an L1 norm as a distance metric, the problem of finding the tightest possible such underestimator *U* can be formulated as follows:
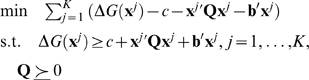
(1)where the decision variables are **Q**, **b**, and *c*, and 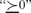 denotes positive semi-definiteness. This problem can be reformulated as a *Semi-Definite Programming* (SDP) problem [30], an important class of convex programming problems [46] which finds many applications in various subjects recently. SDP problems aim at minimizing a linear function subject to the constraints of linear matrix inequalities. Such constraints are nonlinear but convex. Efficient polynomial-time algorithms, such as interior-point algorithms, exist for solving SDP problems. General-purpose solvers are also readily available [47],[48]. We use the callable library of SDPA v6.20 [48] which solves the SDP problem efficiently with a primal-dual interior-point method and exploits the sparsity of the problem. When canonical quadratic underestimators are used as in CGU [26], **Q** is restricted to diagonal matrices without off-diagonal elements. The problem then becomes a Linear Programming (LP) one, which can be regarded as a special case of SDP problems.

There are 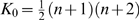 coefficients for *U*, which suggests that the number of samples *K*≥*K*_0_. *K*_0_ equals 10 and 21, respectively, for SDU1 and SDU2. *K* is set to 40 for both SDU methods.

#### Biased sampling

The derived optimal underestimator *U* is exploited to bias further sampling in 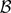. The global minimum of *U* is denoted by **x** *^P^* and referred to as the *predictive conformation*. Since *U* reflects the general structure of the free energy landscape, at least based on the discrete sampling of 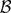, our strategy is to sample in the area around **x** *^P^* such that conformations close to **x** *^P^* are more likely to be selected. This can be achieved by using an acceptance/rejection scheme and the following probability density function (pdf) in 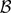:
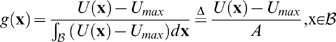
where 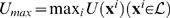.

#### Iteration techniques

The processes of underestimation and biased sampling are iterated with the set of local minima being updated and the search region being gradually shrunk to the neighborhood of predictive conformation **x** *^P^*. Previous samples in 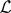 which are energetically unfavorable or too far from **x** *^P^* are be discarded, namely those structures 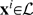 with 
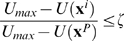
. (*ζ* = 0.7 in this work). Newly sampled structures are added to 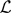 with local minimization starting from **x** *^P^* and 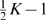 additional biased samples.

We set the convergence criterion based on the proximity of the predictive conformation **x** *^P^* and the current lowest-energy structure **x** *_min_* in 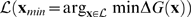. At most 5 iterations are carried on unless convergence is observed earlier, i.e., ∥**x** *^P^*−**x** *_min_*∥≤*ε*. *ε* is set at 1 Å in translations and 0.087 radian (5 degrees) in rotations for SDU1. To approximate equal convergence definition, *ε* is set at 0.1 radian for SDU2. The value of **x** *_min_* at the final iteration provides the final solution for either SDU method.

### Free Energy Evaluation Models

Docking by the SDU algorithms involves the use of two different free energy models. In the rigid body global search the scoring function is
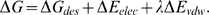
where the desolvation free energy Δ*G_des_* is estimated by the Atomic Contact Potential [49], an atom-level extension of the Miyazawa-Jernigan potential [50], the electrostatic energy Δ*E_elec_* is based on the Coulombic formula with distance-dependent dielectrics ε = 4*r*, and the Van der Waals term Δ*E_vdw_* is adopted from the Charmm potential [51]. The scaling factor *λ*∈[0,1] is dynamically adjusted during the course of the refinement to improve the quality of the underestimator by dampening the effect of the van der Waals term Δ*E_vdw_* and thereby smoothing Δ*G*. Specifically, whenever a “flat” underestimator *U* (this can be determined when the minimum eigenvalue of **Q** is close to zero) is being computed with *λ* = 1, we gradually reduce *λ* with a stepsize of 0.1 until we obtain a more informative underestimator or *λ* reaches 0.

In the flexible local minimization we use the Charmm potential with ε = 4*r*, including the internal energy terms, and perform 100 steps of adopted base Newton-Raphson (ABNR) minimization allowing for side-chain flexibility in the interface [51]. The distance between two components is separately optimized. Specifically, the ligand is pushed towards or pulled apart from the receptor at a stepsize of 0.5 Å along the line segment connecting the two centers of mass. The maximum shift in distance (which defines locality) is 2 Å in this study. Each position is followed by a Charmm minimization described above and energy evaluation. Only non-clashing structures are accepted, judged by the condition Δ*E_vdw_*<0. In fact, we found that PIPER had a higher tolerance toward clashing structures and pushing component proteins closer generally resulted in increased positive Δ*E_vdw_*. To reduce calls to Charmm minimization, in practice we only pull them apart if necessary, i.e., when Δ*E_vdw_*>0 is found. For simplicity we will call the work involved in evaluating ΔG*(**x**) for each conformation **x** a *function evaluation*, although it generally involves several evaluations of Δ*G*(**x**,**s**).

### Quality Measures

As a measure of prediction quality, we select the ligand *C_α_* atoms in the binding site, and calculate the RMSD between their predicted and observed positions. A ligand residue is considered to be in the binding site if any of its atom is within 10 Å of an atom on the receptor. We refer to a structure as near-native (or a “hit”) if its ligand binding site *C_α_* RMSD is less than 10 Å. Although such structures are not really close to the native complex, by rigid body docking it is generally difficult to obtain better results. In fact, since the near-native binding region is selected by cluster size using a clustering radius on the order of 10 Å, the goal of this first step is to generate as many such 10 Å RMSD structures as possible. To show the improvements due to the SDU method, we list the rank of the first cluster that includes a hit, as well as the RMSD between the native structure and the center of the cluster. However, as a more appropriate overall performance measure of the refinement, we also note the number of complexes that have at least one prediction with less than 5 Å RMSD from the native structure in the top 5 clusters.
